# Ecological Succession Pattern of Fungal Community in Soil along a Retreating Glacier

**DOI:** 10.3389/fmicb.2017.01028

**Published:** 2017-06-09

**Authors:** Jianqing Tian, Yuchen Qiao, Bing Wu, Huai Chen, Wei Li, Na Jiang, Xiaoling Zhang, Xingzhong Liu

**Affiliations:** ^1^State Key Laboratory of Mycology, Institute of Microbiology, Chinese Academy of SciencesBeijing, China; ^2^Beijing Radiation Centre, Beijing Academy of Science and TechnologyBeijing, China; ^3^Key Laboratory of Mountain Ecological Restoration and Bioresource Utilization and Ecological Restoration Biodiversity Conservation, Key Laboratory of Sichuan Province, Chengdu Institute of Biology, Chinese Academy of SciencesChengdu, China; ^4^Zoige Peatland and Global Change Research Station, Chengdu Institute of Biology, Chinese Academy of SciencesHongyuan, China; ^5^School of Ecology and Environmental Science, Yunnan UniversityKunming, China

**Keywords:** primary succession, stochastic/deterministic processes, time-dependent, Hailuogou glacier, biogeography

## Abstract

Accelerated by global climate changing, retreating glaciers leave behind soil chronosequences of primary succession. Current knowledge of primary succession is mainly from studies of vegetation dynamics, whereas information about belowground microbes remains unclear. Here, we combined shifts in community assembly processes with microbial primary succession to better understand mechanisms governing the stochastic/deterministic balance. We investigated fungal succession and community assembly via high-throughput sequencing along a well-established glacier forefront chronosequence that spans 2–188 years of deglaciation. Shannon diversity and evenness peaked at a distance of 370 m and declined afterwards. The response of fungal diversity to distance varied in different phyla. Basidiomycota Shannon diversity significantly decreased with distance, while the pattern of Rozellomycota Shannon diversity was unimodal. Abundance of most frequencies OTU2 (*Cryptococcus terricola*) increased with successional distance, whereas that of OTU65 (*Tolypocladium tundrense*) decreased. Based on null deviation analyses, composition of the fungal community was initially governed by deterministic processes strongly but later less deterministic processes. Our results revealed that distance, altitude, soil microbial biomass carbon, soil microbial biomass nitrogen and ${\text{NH}}_{4}^{+}$–N significantly correlated with fungal community composition along the chronosequence. These results suggest that the drivers of fungal community are dynamics in a glacier chronosequence, that may relate to fungal ecophysiological traits and adaptation in an evolving ecosystem. The information will provide understanding the mechanistic underpinnings of microbial community assembly during ecosystem succession under different scales and scenario.

## Introduction

Over the past 100 years, the average global temperature has increased by 0.85°C, which has dramatic consequences for mountain glacier retreating (Stocker et al., 2013). Hence, the global retreat of glaciers is receiving much attention, especially as a signal of climate change (Dyurgerov and Meier, 2000; Oerlemans, 2005; Pelto, 2006). Receding ice cover presenting a chronosequence of development from bare substrate to complex plant communities, glacier forefields are ideal niches for studying primary succession (Nicol et al., 2005). Microorganisms have crucial roles in development of soil, biogeochemical cycling, and facilitating colonization by plants during primary succession (Nemergut et al., 2007; Fierer et al., 2010). Despite their importance, primary succession dynamics of microbial communities and their assembly processes remain comparatively far less understood (Sigler and Zeyer, 2004; Nicol et al., 2005; Bardgett et al., 2007; Nemergut et al., 2007; Schmidt et al., 2008; Bradley et al., 2014; Brown and Jumpponen, 2014; Cutler et al., 2014). As plant colonization is quite slower than that of microbes along a retreating glacier (Schmidt et al., 2014), it is becoming increasingly important to better understand the fundamentals of microbial successional dynamics.

Fungi are one group of the first colonizers of soil and have crucial roles in forming fertile soil that will sustain the growth and development of a complex vegetation community (Fierer et al., 2010; Bradley et al., 2014). Given the unprecedented rate of change in retreating glacier by induced climate change (Stocker et al., 2013), it is necessary to understand how fungal community composition responses to glacier retreating. However, existing results of fungal community succession on retreating glaciers are inconsistent. Several studies have demonstrated that fungal community shifts were tightly linked to the establishment of plants during soil development (Jumpponen, 2003; Zumsteg et al., 2012; Brown and Jumpponen, 2014) and fungal OTU richness increased over successional time (within a century) (Jumpponen et al., 1999; Blaalid et al., 2012; Cutler et al., 2014). Vice versa, other studies indicate that fungal diversity does not respond to distance from glacier terminus (Bradley et al., 2014; Brown and Jumpponen, 2015). This incongruence may be a result of differing successional ages, substrate-associated nutrient limitations, or geographic locations (Brown and Jumpponen, 2015). Therefore, we examine the fungal community spanning two centuries of a retreating glacier, to test whether these community differences are likely to reflect different selection acting along different succession ages.

Glacial retreat which varies in age but has a similar biotic and abiotic history provides an ideal experimental system to study mechanisms of community assembly processes through time and space (Brown and Jumpponen, 2014; Dini-Andreote et al., 2015; Freedman and Zak, 2015). Deterministic vs. stochastic are two types of processes affecting the assembly of microbial communities. Fungal communities have been documented to be determined by deterministic processes, such as pH, salinity, and organic carbon (Fierer and Jackson, 2006; Lozupone and Knight, 2007; Hartman et al., 2008; Nemergut et al., 2011), or by stochastic processes (Peay et al., 2010), or by a combination of both processes (Wu et al., 2013). One study reported that the bacterial community was structured by deterministic processes, while the fungal community was structured by more stochastic processes in primary succession (Brown and Jumpponen, 2014). These findings indicate that communities may be controlled by different ecological processes, which depends on the habitat, community, and spatial scale investigated (Dini-Andreote et al., 2016). More recent studies indicate that time-dependent shifts in the stochastic/deterministic balance occur for microbial communities during secondary succession (Ferrenberg et al., 2013; Zhou et al., 2014; Dini-Andreote et al., 2015). The question rises as to whether stochastic/deterministic balance is time-dependent shift during primary succession.

In this study, a 188-year glacial chronosequence in Hailuogou, China, was selected to investigate the patterns of fungal community succession and test above hypotheses. Our main objectives were to answer the following questions: (1) Do fungal communities exhibit successional trajectories? (2) What are the relative roles of deterministic and stochastic factors in determining community composition and succession? (3) Does the relative importance of deterministic and stochastic factors change over time? The results will provide new insight into the assembly processes of soil fungal communities along retreating glacier soils.

## Materials and methods

### Study site

The Hailuogou glacier (29°24′N, 101°59′E) is located in Mount Gongga, which is the highest peak in the Hengduan Mountain region in the eastern part of and the south-eastern edge of the Tibetan Plateau. As a typical monsoonal temperate glacier, the Hailuogou glacier is the largest in the basin and has a total area of *c*. 25 km^2^ with *c*. 13 km long (Li et al., 2010). Primary vegetation succession has developed on this chronosequence (Li et al., 2010). Mean annual temperature is 4.2°C. The diurnal temperature range is 20.4°C. Mean annual precipitation is 1,947 mm.

### Soil sampling

**Figure 1:**
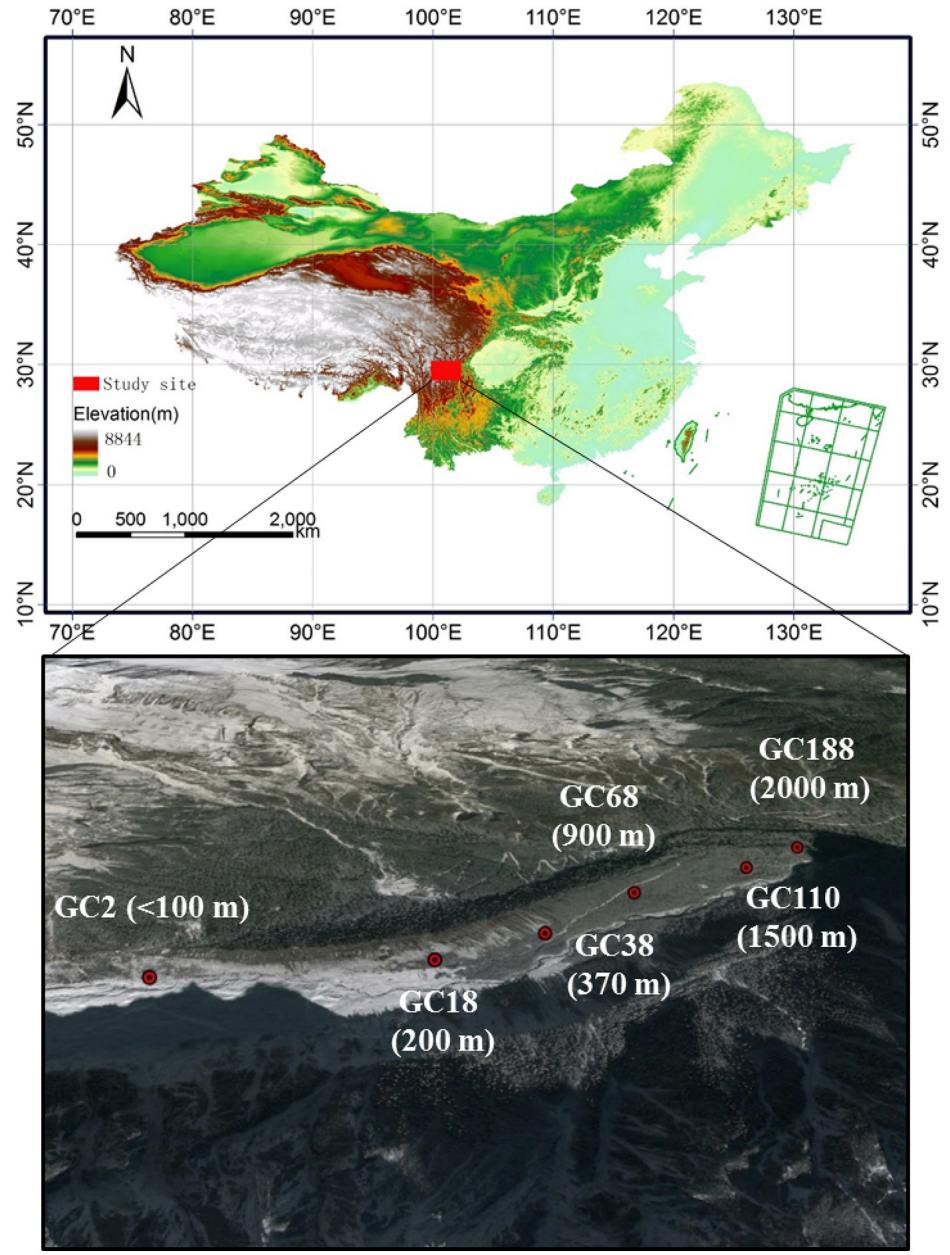
Sampling sites along the ecological succession of the Hailuogou glacier chronosequences. GC represented Glacier Chronosequence. Each stage of succession year and distance from the glacier forefield is specified by letters and a number (GC2: barren, 2 years, <100 m; GC18: 40% vegetation coverage, 18 years, 200 m; GC38, 70% vegetation coverage, 38 years, 370 m; GC68, 75% vegetation coverage, 68 years, 900 m; GC110, 85% vegetation coverage, 110 years, 1,500 m; GC188, 90% vegetation coverage, 188 years, 2,000 m). The dominant vegetation in each stage is listed in Table S1. The horizontal distance from the glacier forefield of sampling sites is in brackets.

Soil samples were collected from six sites along the chronosequence, ranging from <100 to 2,000 m from the glacier terminus (Figure 1). Correspondingly, these sites have deglaciated for *c*. 2, 18, 38, 68, 110, and 188 years, resulting in vegetation that covers 1, 4, 70, 75, 85, and 90% of the surface, respectively. A detailed description of the sampling sites is listed in Table 1 and Table S1. At each site the samples were collected from six replicate plots (10 × 10 m). Each soil sample was immediately placed into a sterile sample bag and sealed, and transported to the lab in an ice-box. One portion was stored at −80°C for DNA extraction while the other half were stored at 4°C for the soil analysis.

**Table 1:** Characteristics of sampling sites, soil properties, and sequencing information in samples from the Hailuogou Chronsequence.

**Site code**	**Succession time (years)**	Horizontal distance ^*^(m)	**Litter thickness (cm)**	**DOC (mg kg^−1^)**	**TN (mg kg^−1^)**	**NH_4_–N (mg kg^−1^)**	**NO_3_–N (mg kg^−1^)**	**SMBN (mg kg^−1^)**	**SMBC (mg kg^−1^)**	**Sequencing coverage**	**Average OTUs number**	**Number of sequences**
GC2	<2	<100	<1	14.5 ± 4.19	751.9 ± 106.6	5.52 ± 0.02	2.6 ± 0.3	3.78 ± 1.72	18.8 ± 3.5	0.97 ± 0.02	78 ± 25	3, 880 ± 1, 683
GC18	18	200	<1	32.6 ± 9.6	324.1 ± 65.4	6.02 ± 0.51	2.9 ± 0.7	6.41 ± 2.61	35.9 ± 14.6	0.96 ± 0.01	66 ± 30	3, 214 ± 1, 806
GC38	38	370	5	43.8 ± 1.5	648.9 ± 17.9	7.82 ± 0.62	20.6 ± 6.0	11.3 ± 4.60	60.1 ± 24.6	0.94 ± 0.00	90 ± 21	3, 366 ± 1, 101
GC68	68	900	10	122.2 ± 54.4	968.0 ± 52.1	9.35 ± 0.73	24.8 ± 10.3	30.7 ± 12.5	116.4 ± 47.5	0.94 ± 0.02	81 ± 27	5, 521 ± 1, 001
GC110	110	1,500	15	629.8 ± 266.2	1, 349.1 ± 269.7	53.6 ± 20.4	2.2 ± 0.3	101.6 ± 41.5	506.9 ± 206.9	0.96 ± 0.01	67 ± 24	7, 011 ± 1, 033
GC188	188	2,000	17	523.1 ± 187.1	1, 980.1 ± 190.3	79.8 ± 29.4	6.9 ± 1.2	99.2 ± 40.5	437.9 ± 178.6	0.96 ± 0.02	81 ± 24	4, 487 ± 2, 002

*Presented values of soil properties are mean ± SD (samples per group n = 6). DOC, Dissolved organic carbon; TN, Total nitrogen; NH_4_–N, Ammonia; NO_3_–N, Nitrate; SMBN, Soil microbial biomass nitrogen; SMBC, Soil microbial biomass carbon*.

* *Horizontal distance refers to the distance to the end of glacier*.

### Soil physiochemical analysis

The pH-value was determined at a soil to water ratio of 1:5 (wt/vol). Total nitrogen (TN) was determined using a modified Kjeldahl method. Ammonium (${\text{NH}}_{4}^{+}$–N) and nitrate (${\text{NO}}_{3}^{-}$–N) were measured using a continuous flow analyzer (Skalar SA 1000, Breda, The Netherlands). The soil samples for analysis of the dissolved organic carbon (DOC) were prepared by mixture with 0.5 M K_2_SO_4_ in a centrifuge tube, then shaking for 1 h on a reciprocal shaker and centrifuging at 13,000 rpm for 30 min at 4°C. The supernatant was filtered through a 0.45-μm glass fiber filter. All the extracts were analyzed by a continuous flow analyzer of San++ (Skalar SA 1000, Breda, The Netherlands).

Soil microbial biomass carbon (SMBC) and soil microbial biomass nitrogen (SMBN) were determined by the chloroform fumigation extraction method after 14 days of conditioning at 50% of their total water holding capacity under 25°C. This was followed by a 0.5 M K_2_SO_4_ extraction method for both the non-fumigated and fumigated samples (Brookes et al., 1985; Vance et al., 1987). The SMBC extraction was analyzed by a TOC-V WP (SHIMADZU, Japan) and the SMBN extraction was digested and then analyzed by a San++ continuous flow analyzer (Skalar SA 1000, Breda, The Netherlands).

### DNA extraction, PCR, and pyrosequencing

Total DNA for the metabarcoding analyses was extracted from *c*. 0.5 g of frozen soil with the UltraClean Soil DNA extraction kit (MoBio, Carlsbad, USA), according to the manufacturer's instructions. The obtained DNA was quantified with a NanoDrop device and stored at −20°C. An aliquot of 50-ng purified DNA from each sample was used as template for amplification. A set of primers was designed by adding a Roche 454 “A” pyrosequencing adapter, and a unique 8-bp barcode sequence to the forward primer ITS1F (5′-CTTGGTCATTTAGAGGAAGTAA-3′) and the reverse primer ITS2 (5′-GCTGCGTTCTTCATCGATGC-3′) (White et al., 1990) to generate PCR ITS1 region fragments of *c*. 400 bp. Each sample was amplified in triplicate with 50 μl reactions under the following conditions: 94°C for 4 min, 30 cycles of 30 s at 94°C (denaturation), 50°C for 1 min (annealing), and 72°C for 90 s (extension), followed by 10 min at 72°C. PCR products were pooled together and purified by the Agarose Gel DNA purification kit (TaKaRa, China) and quantified with the NanoDrop device. Samples were run on a ROCHE 454 FLX+ platform (Roche, Basel, Switzerland) at the National Human Genome Center of China at Shanghai, China, according to the manufacturer's instruction manual. These raw data are deposited in the NCBI Sequence Read Archive with the accession number SRP093928.

### Sequence analysis

The raw sequence data were de-multiplexed using the QIIME toolkit (Caporaso et al., 2010). Reads with a quality score of less than 20 were filtered out (split_libraries_fastq.py; QIIME) (quality score <20, homopolymer runs of >6 nt and length <200 nt), and the remaining reads were assigned to samples according to their corresponding barcode. The extracted sequences were binned into operational taxonomic units (OTUs) at 97% identity using UPARSE (Edgar, 2013); species-level similarity was denoted by a closed-reference OTU that used the UNITE ITS database (Abarenkov et al., 2010) as a reference (pick_otus.py; QIIME). One representative of each OTU was selected for the downstream analyses, and taxonomy was assigned to each representative (pick_rep_set.py, assign_taxonomy.py; QIIME). The α-diversity of each sample was calculated using the Shannon metric (alpha_diversity.py; QIIME).

All singleton OTUs from the downstream analyses were excluded because whether singleton data represents true diversity or sequencing errors is an unsettled issue (Tedersoo et al., 2010). Evenness and Shannon diversity indices (*H*′) were estimated for total fungi as well as their most abundant taxa based on OTU abundance matrices rarefied to the lowest sequence numbers. Chao and ACE were calculated at the 97% threshold. Sampling effort was estimated using Good's coverage.

### Statistical analyses

To evaluate the effects of distance from the glacier terminus on OTU richness, Shannon diversity, and evenness, we fitted linear and cubic regressions. In these analyses we treated distance from the glacier terminus as a continuous (linear, cubic regression). The effect of distance on the relative abundances of the most abundant and frequency taxa were assessed by linear and quadratic regressions. All statistical analyses were performed in R.

Principal coordinate analyses (PCoA) were used to compare dissimilarities among the fungal communities across succession distance, as based on the Bray-Curtis dissimilarity. To determine the relationship between succession distance and Bray-Curtis distances, scores for the first two principal coordinates against succession distance were regressed. Mantel test is multivariate correlation analysis by means of comparison between dissimilarity matrices of same dimension. Mantel tests were performed to examine community turnover along environmental and succession gradients. The overall effect of glacier retreating on fungal community was determined using and permutational MANOVAs (PERMANOVAs). These above analyses were performed in the *vegan* package in R.

β-diversity is useful to understand patterns of species diversity across various spatial scales and provides critical insights into the role of deterministic and stochastic processes in shaping community compositions and structure (Chase, 2007, 2010; Chase and Myers, 2011; Zhou et al., 2014). Low dissimilarity among communities that are otherwise identical in environmental conditions would imply a predominant role for deterministic assembly, whereas high dissimilarity would suggest a large role of stochastic assembly (Chase, 2007, 2010). However, the measures of β-diversity are dependent on both α- and γ-diversity (Chase et al., 2011). It is not clear whether a change in β-diversity is due to the differences in the underlying assembly processes that generate β-diversity or to the differences in α- and γ-diversity (Chase et al., 2011). β-Diversity null deviation approach uses a null model to create stochastically assembled communities from the regional species pool to determine the degree to which the observed β-diversity patterns deviate from stochastic assembly(Chase and Myers, 2011; Tucker et al., 2016). Hence, this approach can assess changes in β-diversity that result from the relative influences of deterministic and stochastic processes, and not from changes in α-diversity. We measured the null deviation as the relative difference of the observed β-diversity from the null-model β-diversity, (β_obs_−β_null_)/β_null_, where β-diversity was measured as the Sorenson-Czekanowski dissimilarity. For each sample, the expected β-diversity under the null model was calculated from 999 stochastically assembled communities. The γ-diversity was calculated from the total number of species detected in the “species pool” from all sites. To test for treatment differences in the null deviation, we conducted permutation tests by first randomly permuting treatment labels, then re-simulating the null models and re-calculating the null deviations for each of the 999 permutations.

Standard effect size (SES), another similar quantitative index, was introduced into this study to measure the influence of deterministic factors on community composition and abundance over time. β-Deviation was calculated as the observed β-diversity subtracted the mean of the null distribution of β-diversity values, then divided by the standard deviation of this distribution (Kraft et al., 2011).

## Results

### Distribution of taxa and phylotypes

After quality control (QC), 52% of fungal ITS-1 sequences were removed through each step of our pipeline and only 145,450 sequences from 299,945 were remained (Table S2). Sequence loss through each step of our pipeline is summarized in Table S2. Clustering of the sequences resulted in a total of 611 OTUs. The coverage of each sample ranged from 0.92 to 0.99 (Table 1), suggested that these libraries detected a large majority of the fungal diversity in the samples used in our study. Soil fungal communities were strongly dominated by the diverse Ascomycota and Basidiomycota (47.0 and 48.3% of total sequences, respectively). Surprisingly, a relatively higher component of Rozellomycota (Cryptomycota) was detected for 4.49% of total sequences and 38 OTUs (6.2%), compared with that of Chytridiomycota and Glomeromycota, which was constituted of 0.2 and 0.01% of the total sequences, respectively. The sequencing data suggested that the fungal community was uneven and that 10 dominant OTUs (abundance >1%) out of the 611 OTUs accounted for 70% of the total reads. Among those 10 OTUs, three most abundance OTUs were assigned to *Cysrodendron* sp. (25.5% of total sequences), and followed by *Cryptococcus terricola* (16.4%), and *Scleroderma areolatum* (15.9%).

A positive linear relationship was found between the relative abundance of Rozellomycota and nitrate concentration (Linear regression, *R*^2^ = 0.34, *P* < 0.001). No significant relationship was detected between nitrate concentration and Ascomycota and Basidiomycota.

### Dynamics of fungal diversity and community along the primary succession gradient

**Figure 2:**
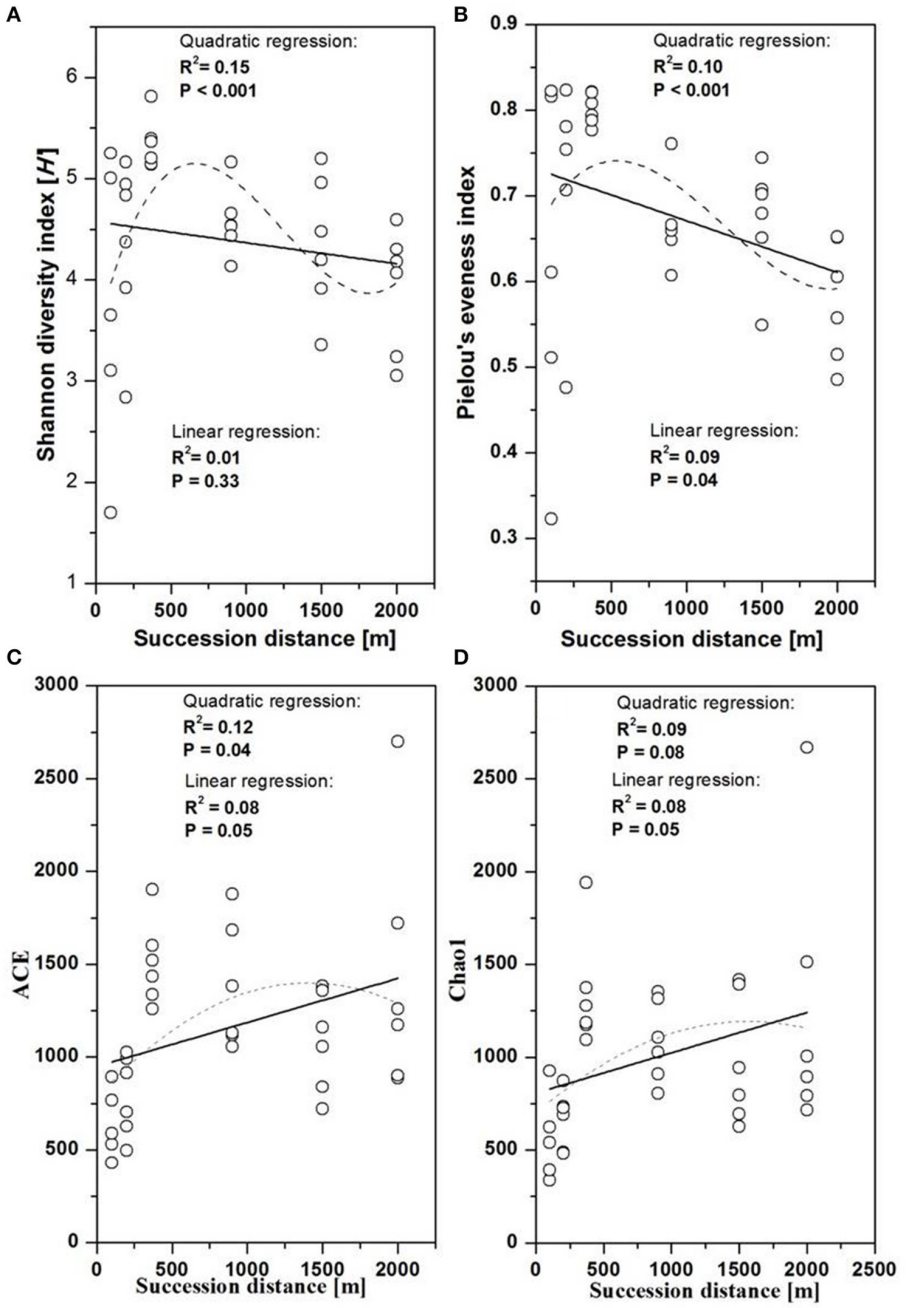
Changes in fungal diversity as a function of succession distance. Linear and quadratic regression of fungal **(A)** Shannon diversity, **(B)** evenness, **(C)** Chao1 richness, and **(D)** ACE along the chronosequence.

**Figure 3:**
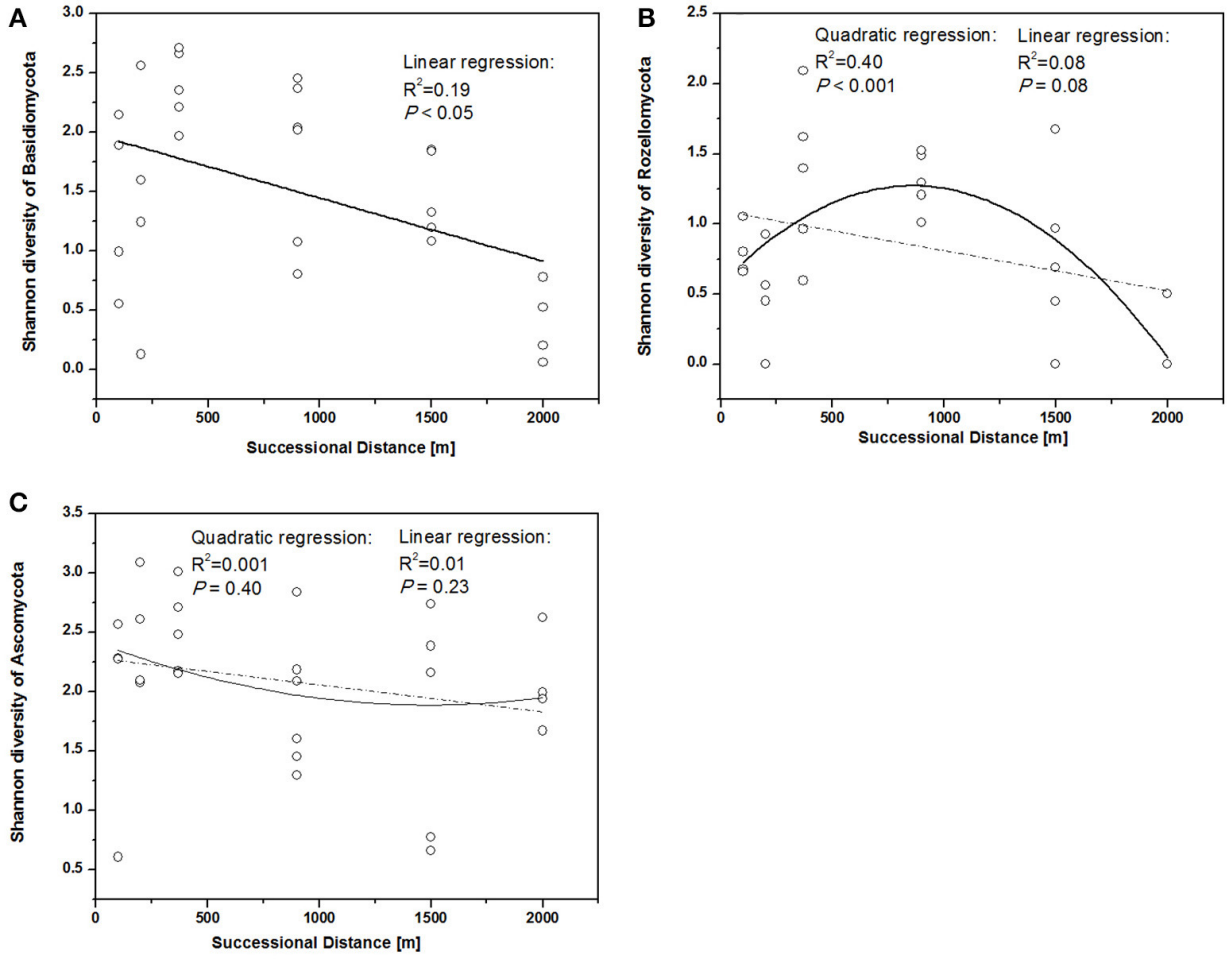
Shannon diversity of different fungal phyla along different successional distance. Linear and quadratic regression of **(A)** Basidiomycota, **(B)** Rozellomycota, and **(C)** Ascomycota along the chronosequence.

The fungal indices for Shannon diversity and evenness showed unimodal patterns over distance from the glacier terminus (Figures 2A,B), while Chao1 richness and ACE showed no significant response trend (Figures 2C,D). Shannon diversity and evenness peaked at a distance of 370 m and declined afterwards. The Shannon diversity index of Basidiomycota significantly decreased with distance (Figure 3A, linear regression, *P* < 0.05), while the pattern of Rozellomycota diversity response to succession distance was unimodal (Figure 3B, quadratic regression, *P* < 0.05). No significant response pattern was detected between Ascomycota diversity and succession distance (Figure 3C, linear regression and quadratic regression, *P* > 0.05).

**Figure 4:**
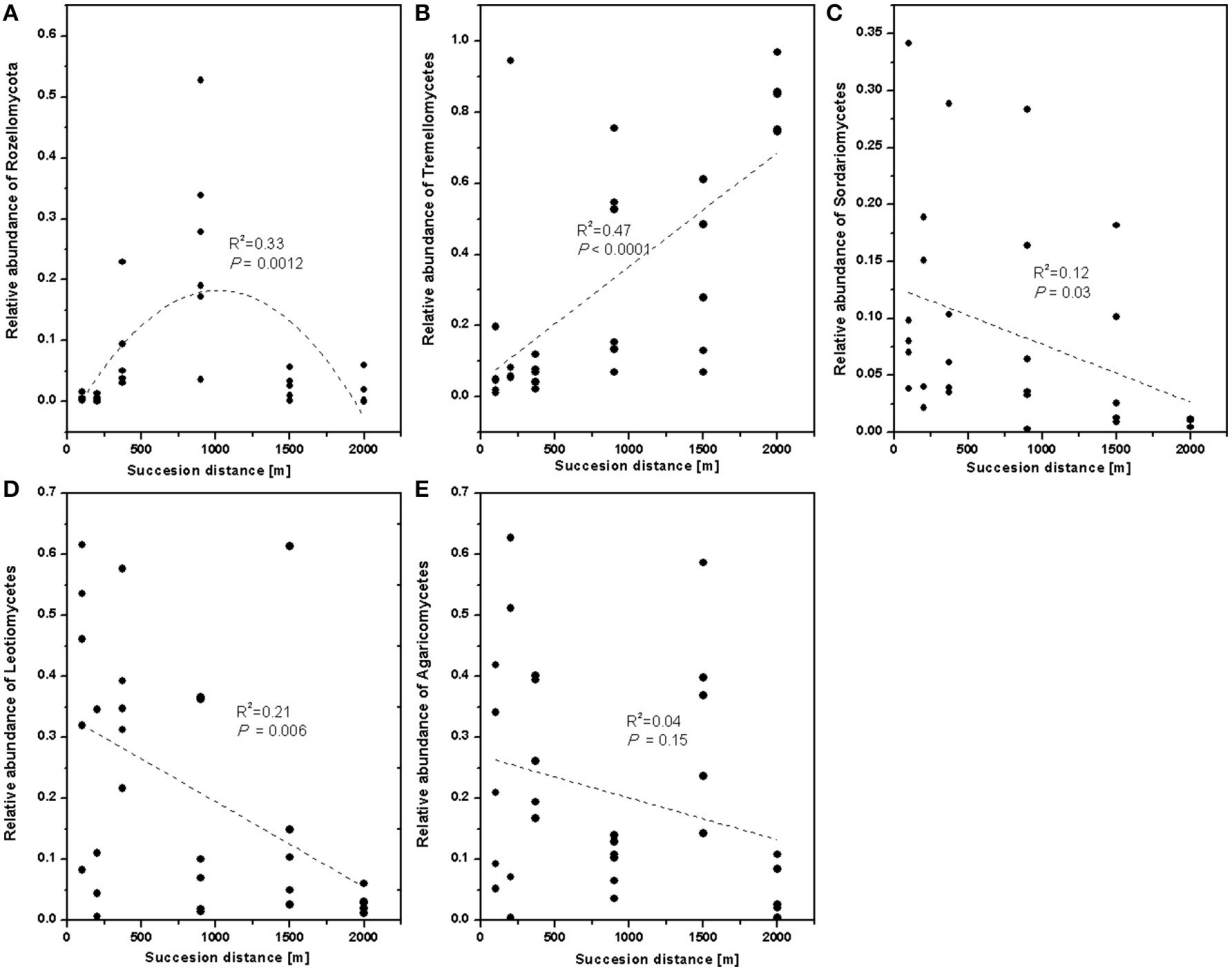
The relative abundance of fungal class along the successional distance. Linear and quadratic regression of **(A)** Rozellomycota, **(B)** Tremellomycetes, **(C)** Sordariomycetes, **(D)** Leotiomycetes, and **(E)** Agaricomycetes along the chronosequence. The trends along the succession distance were indicated by dash lines.

To verify the differences observed in the fungal communities along chronosequence, the relative abundances of the different classes were compared. The relative abundance of Rozellomycota (Cryptomycota) significantly increased in younger developing soil but declined after 900 m (Figure 4A). The relative proportion of Tremellomycetes increased (Figure 4B), whereas those of Sordariomycetes (Figure 4C), and Leotiomycetes decreased with distance (Figure 4D). Agricomycetes did not significant response to distance (Figure 4E).

**Figure 5:**
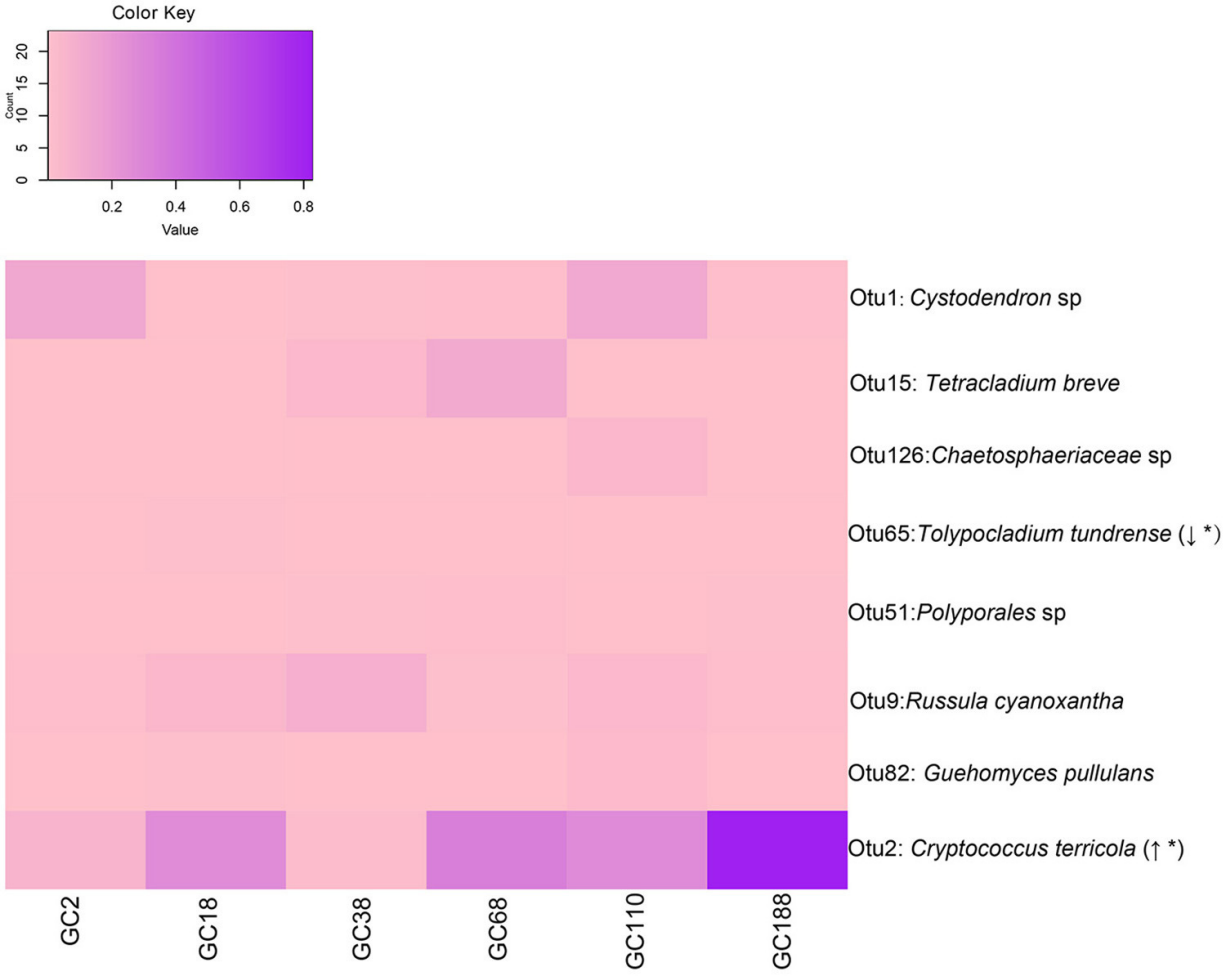
Fungal OTUs occurred in all sites and they changed in frequency with successional age (i.e., distance from the glacier terminus). Italic type refers to the best BLASTn match (nr/nt) with the exclusion of uncultured/environmental samples. Linear regression of abundance of those OTUs along the chronosequence. Direction of change indicates if the abundance of OTUs increased (↑) or decreased (↓) across the distance from the glacier. Asterisk (^*^) indicates statistical significance. The relative abundance of OTUs are depicted by color intensity, with the legend indicated at the top of the figure. The increasing color intensity indicated a higher relative abundance.

Ecosystem processes and successional dynamics are probably driven by those community members that occur most frequently. There were eight OTUs that occurred at all the six sites (Figure 5). Interestingly, the relative abundance of OTU2 (*C. terricola*) significantly increased with increased distance from the glacier terminus (Linear regression, *P* < 0.001; Figure S1), but its relative abundance was negatively related to fungal diversity (Figure S2). However, the relative abundance of OTU65 (*Tolypocladium tundrense*) decreased with distance from the glacier terminus (Linear regression, *P* < 0.001).

**Figure 6:**
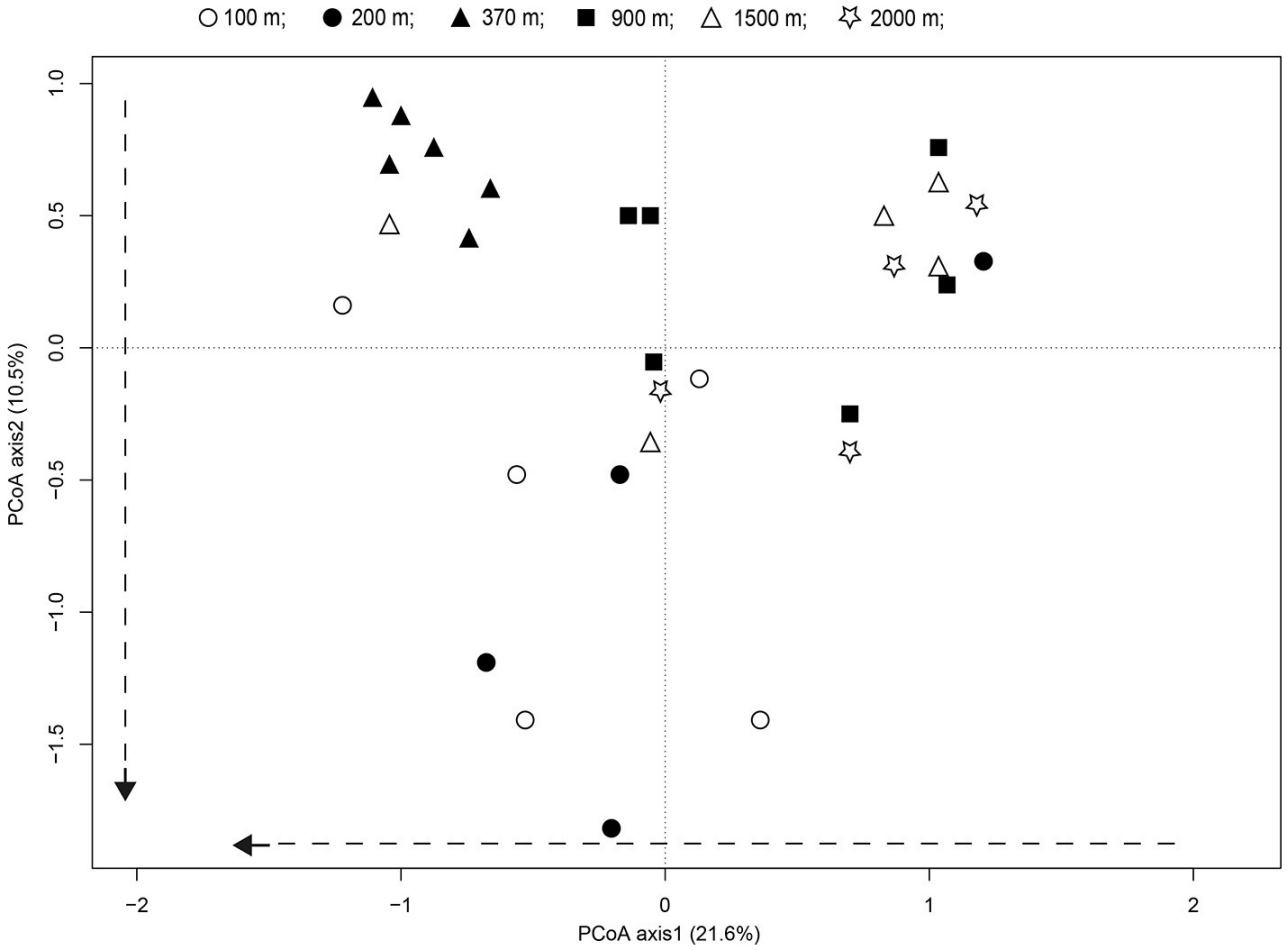
Patterns of fungal communities across successional distance in the glacier chronosequence. Principal coordinates analysis PCoA representing the overall variability in fungal communities. Variance explained by each PCoA axis is given in parentheses. The PCoA of the fungal community showed strong successional trajectories, with dashed arrows representing the direction of community shifts as indicated by significant linear regression statistics.

Principal coordinate analyses (PCoA) and PERMANOVA analyses revealed significant differences in the fungal communities along the successional gradient (Figure 6, PERMANOVA, pseudo-F = 3.82, *P* < 0.001). The fungal communities from younger soils (distance: <100 and 200 m) were significantly differentiated to samples from distance of 370 m, indicated the soils are developing. Whereas, the communities from the older soils (distance: 900–2,000 m) were more similar to each other, indicated the soils are developed (Figure 6). The scores for axis 1 representing 21.6% of the variability increased substantially along with distance from the glacier terminus (*t* = 2.43, *P* < 0.05). Treating distance from the glacier as a categorical variable, the axis 1 scores differed among distance classes [ANOVA: *F*_(1, 32)_ = 5.89, *P* < 0.05]. This was mainly attributable to the axis 1 scores between the younger soils and older soils (Tukey's HSD pairwise comparison at an alpha level = 0.05). The scores for axis 2 representing 10.5% of the variability also tended to increase with distance from the glacier terminus (*t* = 2.59, *P* = 0.02).

Mantel test results showed that distance (*r* = 0.26, *P* = 0.003), altitude (*r* = 0.19, *P* = 0.02), SMBN (*r* = 0.37, *P* = 0.016), SMBC (*r* = 0.34, *P* = 0.016), and ${\text{NH}}_{4}^{+}$–N (*r* = 0.34, *P* = 0.06) correlated with fungal community.

### Community assembly processes

**Figure 7:**
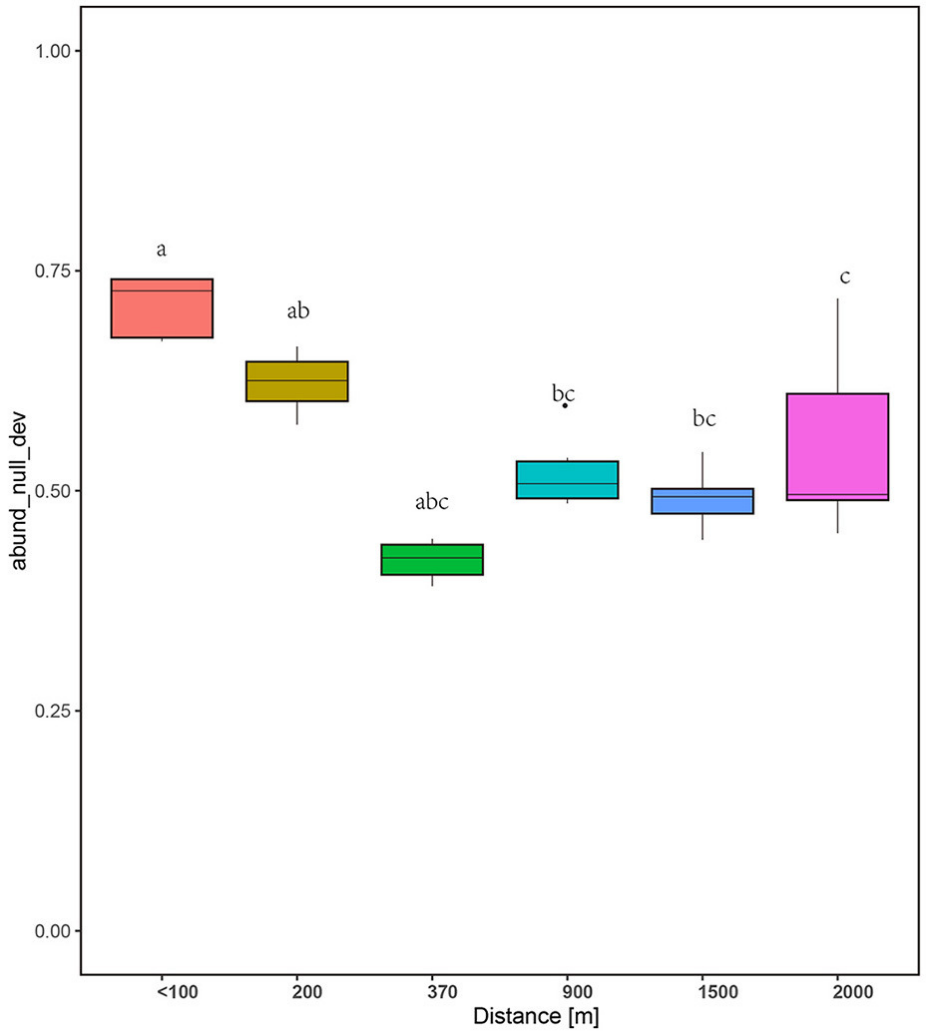
Plots showing the null deviation of fungal communities along succession age. A null deviation close to zero suggests that stochastic processes are more important in structuring the community, whereas larger positive or negative null deviations suggest that deterministic processes are more important.

The null deviation approach can provide powerful insights into community assembly mechanisms (Chase and Myers, 2011). A null deviation close to zero suggests that stochastic processes are more important in shaping community structure, whereas higher positive or negative null deviations suggest that deterministic processes are more important (Chase, 2007, 2010; Ferrenberg et al., 2013). The relative contribution of both processes was dynamic along the successional stages tested (Figure 7). In the initial stage, the fungal communities significantly deviated from the stochastic assembly model (relative null deviation = 0.67–0.74) more than in later stages (relative null deviation = 0.45–0.52) (*P* < 0.05). Importantly, the intermediate stages and later stages showed a relatively consistent deviation from the stochastic assembly model, with no significant changes in the null deviation value between samples (Figure 7). Also, SES, which is used to measure the influence of deterministic factors on community composition and abundance (Kraft et al., 2011), was higher for the communities at the early stages than intermediate and later stages (Figure S3).

## Discussion

High-throughput sequencing technologies now permit us to study mechanisms that govern community assembly processes through time and space (Brown and Jumpponen, 2014, 2015; Cutler et al., 2014). Here, we reported the fungal community presented in different developing soils formed after the retreat of an alpine glacier. Fungal diversity and community significantly responded to the glacier-retreating chronosequence. The highest fungal diversity was found in the intermediate age of the retreating glacier but decreased afterwards, which follows a similar pattern seen for plants and animals (Jones and Henry, 2003; Hodkinson et al., 2004). This may have resulted from a significant unimodal pattern of plant diversity in same study sites (Li and Xiong, 1995). Fungal diversity was strongly correlated to plant diversity (Peay et al., 2013). Different plant taxa provide diverse quantity and quality of nutrients via plant root exudates and litter decomposition (McGuire et al., 2012). Hence, diverse mixtures of plant may lead to diverse fungal taxa via resource partitioning (McGuire et al., 2010).

At a later stage, the ecosystem maturation resulted in a decline in diversity, probably due to domination of the stronger competitors (Blaalid et al., 2012). An extremely high abundance of *C. terricola* (OTU2), significantly negatively correlated to fungal diversity (Figure S2), lends evidence to supported this viewpoint. Yeast-dominated systems have been reported on many glacier and snow surfaces (Branda et al., 2010; Brown and Jumpponen, 2014; Cutler et al., 2014; Brown et al., 2015). In the present study, OTUs that represented yeasts were dominant across all the sampling sites and accounted for 18–90% of the frequencies (Figure S1). The budding growth lifestyle and expanding stress tolerance genes make yeasts to be stress-tolerant fungi (Smith et al., 1992; Whiteway and Bachewich, 2007; Su et al., 2016) and be selected by harsh environments (Treseder and Lennon, 2015). Furthermore, *Cryptococcus* sp. possesses polysaccharide capsule which helps to keep the water intracellularly and to adapt the low water availability and temperature environment (Zaragoza et al., 2006; Treseder and Lennon, 2015). Tolerant microbial groups might lead to soil C accumulation via their production of recalcitrant C residues in soil (Treseder and Lennon, 2015), which should result in organic matter accumulation in retreating glacier fronts.

Rozellomycota is a newly described division of fungi (James et al., 2006; Jones et al., 2011) that is distributed in many habitats (Jones et al., 2011; Lazarus and James, 2015). Although the ecology of Rozellomycota is less understood (Corsaro et al., 2014; Grossart et al., 2016), single cell lifestyle supports their occurrence in unusual niches. Consistence with the results of Dini-Andreote et al. (2016), a high abundance of Rozellomycota (*c*. 8.8%–25.6%) was detected in the intermediate stage (370 and 900 m). In addition, a positive linear relationship was found between the relative abundance of Rozellomycota and nitrate concentration (*R*^2^ = 0.34, *P* < 0.001), which suggests that nitrate may be one factor shaping Rozellomycota's global distribution (Tedersoo et al., 2014).

A dynamic pattern of fungal community assembly processes is determined by deterministic and/or intrinsic selection over time. During the initial stages of succession, environmental filtering (deterministic process) had a greater influence on structuring the fungal communities, a result consistent with Rime et al. (2015) reported on fungal community and with work from animal and plant communities (Schlegel and Riesen, 2012). This greater determinism may partly arise because environmental factors operate as the driving factors that first influence the species arriving on site (Schlegel and Riesen, 2012). Slight differences in the colonization (intrinsic) taxa in the initial communities could thus alter successional trajectories. Yeast dominated at our study site, whereas Brown and Jumpponen (2014) reported that stochastic processes governed fungal community assembly in the initial stage with *Mortierella* dominant. In early stages of succession, microbial communities are sustained by ancient and recalcitrant carbon resources (Bardgett et al., 2007), which are vulnerable and largely influenced by soil moisture that then affects fungal community structure (Zumsteg et al., 2012, 2013; Rime et al., 2015). Due to yeast's budding growth lifestyle (Whiteway and Bachewich, 2007), they must obtain resources from the microenvironment that immediately surrounds them (Treseder and Lennon, 2015). Thus, even a slight shift in these microenvironment resources is likely to have strong effects on yeast colonization and growth (Treseder and Lennon, 2015). In contrast, *Mortierella* as filamentous fungi do not have this restriction, since they can forage over relatively long distances—up to several meters for some species (Boddy, 1999).

The deterministic process became less important in the intermediate and late stages of succession of the retreating glacier than in the initial stages. One possible explanation may relate to nutrients availability. As plants become established during soil development in the intermediate and late stages, the local plant species take on a major role in modulating the fungal communities by the progressive buildup of C-rich sources (Knelman et al., 2012). In particular, plant type can influence a fungal community by altering the availability of soil nutrients (Yuan et al., 2014). However, our results suggest that the magnitude of nutrients provided by dominant vegetation changes did not exceed real effects, thus generating a relatively lowered fungal turnover during the intermediate and late stages. Another explanation may link to larger intraspecific variations of fungal species (Debaud et al., 1999). In the later stage, the niche range is much wider for same fungal species with distinct traits. Hence, taxa, species, or clades that are abundant may have a larger chance of evolving into several different specialist clades and thereby occupy several different habitats due to local adaptations, but still belong to the same taxa (Östman et al., 2010). Most abundant fungal species were widespread in later stage (Figure 5) that also supported this viewpoint. Hahn and Pockl (2005) reported that bacterial taxa with identical 16S rRNA sequences comprise several distinct ecotypes.

In conclusion, the dynamics of fungal communities along a primary successional chronosequence were strongly affected by distance from glacier terminus. The yeast-dominated system in retreating glacier indicates that the sampling sites are still stressful, even after 188 years of development. These results also suggest that the edaphic properties during early succession have led to strong selection in the fungal species' abundances. The rate of fungal turnover was higher in the early succession but slowed down afterwards, indicating that locally abundant taxa became more dominant over time. Deterministic processes was a major driving mechanism in early successional sites that shaped the fungal communities and selected the members to successfully establish and survive. The dynamics of the assembly process revealed in this study suggest that multiple processes may govern fungal community establishment in different glacier soils. Together, our results highlight the importance of dispersal limitation and environmental filtering as ecological forces, which together, act to shape community composition of soil fungal communities in retreating glacier.

## Author contributions

NJ, HC, JT, and XL: conceived and designed the experiments. JT, YQ, WL, and BW performed the experiments. JT and XZ analyzed the data. JT, YQ, NJ, HC, and XL wrote the manuscript. All of the authors assisted in writing the manuscript, discussed the results, and commented on the manuscript.

### Conflict of interest statement

The authors declare that the research was conducted in the absence of any commercial or financial relationships that could be construed as a potential conflict of interest.
