# Supplementary material for: Ecological Succession Pattern of Fungal Community in Soil along a Retreating Glacier
Source: Front Microbiol. 2017 Jun 9;8:1028. doi: 10.3389/fmicb.2017.01028 (PMC5465267; doi:10.3389/fmicb.2017.01028)
Supplement: Supplementary file 1 [file Table1.DOCX]

| Site code | Latitude | Longitude | Elevation (m) | Dominant vegatation |
| --- | --- | --- | --- | --- |
| GC2 | 101°59′33″N | 29°34′02″W | 2950 | Barren soil |
| GC18 | 101°59′37″N | 29°34′06″W | 2947 | *Salis* spp., *Hippophae rhamnoides*, *Populus yunnanensis*, *Betula utilis* |
| GC38 | 101°59′51″N | 29°34′12″W | 2920 | *Salis* spp., *Hippophae rhamnoides*, *Populus yunnanensis*, *Abies fabri* |
| GC68 | 102°00′04″N | 29°34′22″W | 2924 | *Salis* spp., *Hippophae rhamnoides*, *Populus yunnanensis*, *Sorbus* spp., *Prunus trichostoma*, *Lonicera japonica*, *Acer laxiflorum* |
| GC110 | 102°00′21″N | 29°34′29″W | 2906 | *Populus yunnanensis*, *Betula utilis*, *Rhododendron* spp., *Sorbus* spp., *Prunus trichostoma*, *Lonicera japonica*, |
| GC188 | 102°00′30″N | 29°34′35″W | 2880 | *Populus yunnanensis*, *Betula utilis*, *Rhododendron* spp., *Sorbus* spp., *Prunus trichostoma*, |

TableS1 Characteristics of sampling sites of the Hailuogou Chronsequence
