# Supplementary material for: Ecological Succession Pattern of Fungal Community in Soil along a Retreating Glacier
Source: Front Microbiol. 2017 Jun 9;8:1028. doi: 10.3389/fmicb.2017.01028 (PMC5465267; doi:10.3389/fmicb.2017.01028)
Supplement: Supplementary file 2 [file Table2.DOCX]

Table S2: Sequences lost in quality control pipeline.

| Command | Num. Sequences | % Removed |
| --- | --- | --- |
|  | 299,945 | - |
| Trimming | 193,360 | 36 |
| Filter | 175,506 | 41 |
| Discard Singleton | 145,450 | 52 |
| Final |  | 52 |
