# Supplementary material for: Ecological Succession Pattern of Fungal Community in Soil along a Retreating Glacier
Source: Front Microbiol. 2017 Jun 9;8:1028. doi: 10.3389/fmicb.2017.01028 (PMC5465267; doi:10.3389/fmicb.2017.01028)
Supplement: Supplementary file 3 [file Image1.PDF]

Figure S1 The relative abundance of OTU2 (i.e *Cryptococcus terricola*) along successional age (distance from the glacier terminus)

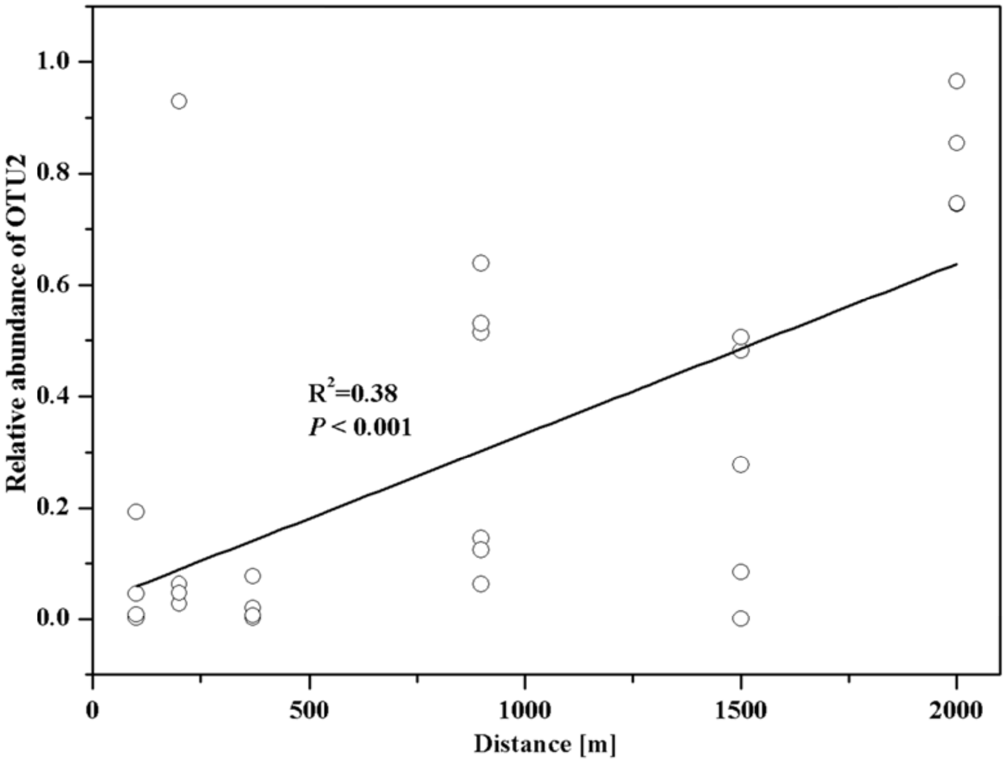

#
