# Supplementary figures and images for: Ecological Succession Pattern of Fungal Community in Soil along a Retreating Glacier
Source: Front Microbiol. 2017 Jun 9;8:1028. doi: 10.3389/fmicb.2017.01028 (PMC5465267; doi:10.3389/fmicb.2017.01028)

Figure S2 The relationship between OTU2 abundance and fungal diversity

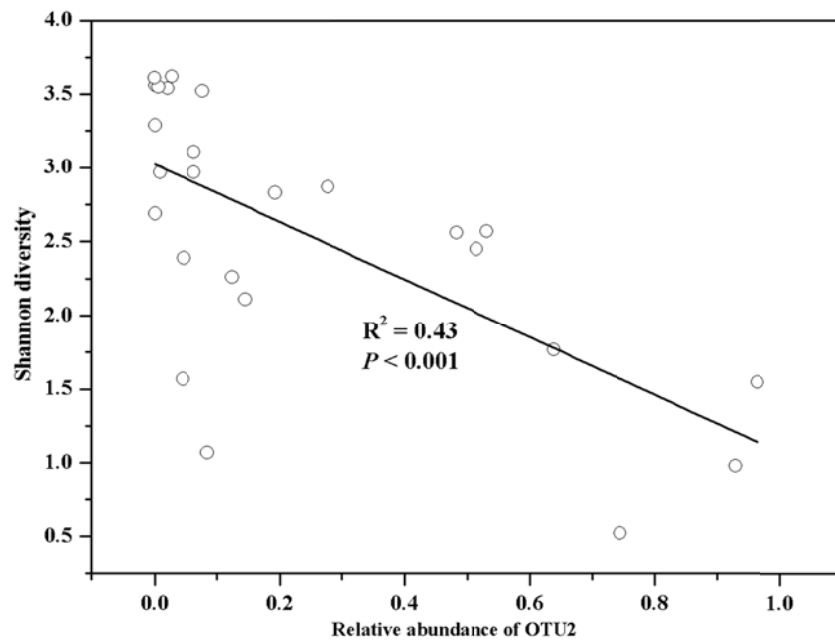

#

Supplement: Supplementary file 4 [file Image2.PDF]
