# Supplementary material for: Ecological Succession Pattern of Fungal Community in Soil along a Retreating Glacier
Source: Front Microbiol. 2017 Jun 9;8:1028. doi: 10.3389/fmicb.2017.01028 (PMC5465267; doi:10.3389/fmicb.2017.01028)
Supplement: Supplementary file 5 [file Image3.PDF]

Figure S3 Changes of standard effect size (SES) during the succession of the fungal community. SES is used to measure the influence of deterministic factors on community composition and abundance. Negative values indicating less β-diversity than expected under the null model

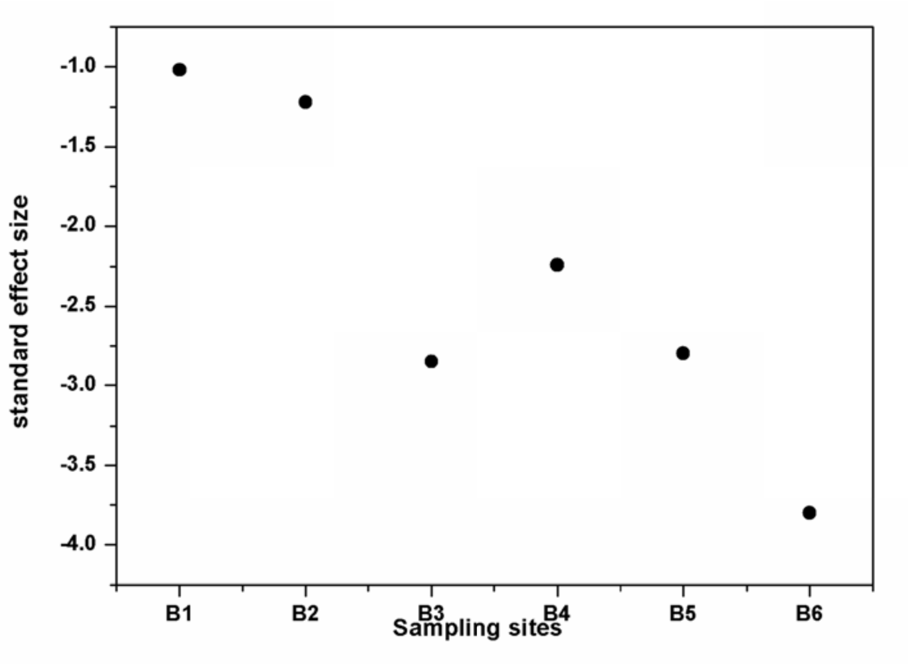

#
